# Practical Reasoning and Practical Argumentation: A Stakeholder Commitment Approach

**DOI:** 10.1007/s11245-023-09901-w

**Published:** 2023-04-03

**Authors:** Kees van Berkel, Jean H. M. Wagemans

**Affiliations:** 1 Institute for Philosophy II, Ruhr Universität Bochum, Bochum, Germany; 2 Amsterdam Center for Language and Communication (ACLC), University of Amsterdam, Amsterdam, The Netherlands

**Keywords:** Commitments, Deliberation, Practical argumentation (PA), Practical reasoning (PR), Stakeholder commitment approach (SCA)

## Abstract

This paper examines the conceptual and terminological overlap between theories and models of practical deliberation developed within the fields of Practical Reasoning (PR) and Practical Argumentation (PA). It carefully delineates the volitional, epistemic, normative, and social commitments invoked and explicates various rationales for attributing the label ‘practical’ to instances of reasoning and argumentation. Based on these analyses, the paper develops a new approach to practical deliberation called the Stakeholder Commitment Approach (SCA). By distinguishing between ‘problem holder’ and ‘problem solver’, and specifying the distributions of attributable commitments among the stakeholders, the SCA introduces an extension and refinement of the grounds for assigning the label ‘practical’ that brings PR and PA closer together.

## Introduction

Philosophers in the fields of Practical Reasoning (PR) and Practical Argumentation (PA) have developed a variety of theories and models of deliberation as a means for resolving so-called ‘practical’ problems. These are problems expressed in questions such as: ‘Should I buy this house?’, ‘What can you do to be on time for your job interview?’ and ‘What is the best way to improve the situation of precarious groups in society?’ Within both fields, practical deliberation is usually described in terms of the specific premises leading to a conclusion about an action and the various types of commitments attributed to the agent(s) (supposedly) performing that action. As a result, the theories and models developed within PR and PA of how people address practical problems—through reasoning, group deliberation, and argumentation—show considerable conceptual and terminological overlap.

Apart from these similarities, one can also identify some crucial differences. In the field of PR, for example, various cases of reasoning aimed at resolving practical problems are often labeled ‘theoretical reasoning’ rather than ‘practical reasoning’. An example is the following inference resulting in advice to a second party:

To be on time for your job interview, you should take the bike because that is the fastest means of transportation in Amsterdam.

Other examples of what counts as ‘theoretical’ within PR are inferences expressing the benefits or consequences of certain actions and the specific conditions for carrying them out. Within PA, in contrast, such inferences often count as ‘practical’ because they are an integral part of a proposal for action rather than a theoretical hypothesis about reality.

In this paper, we analyze prominent theories and models developed within the fields of PR and PA, focusing on their procedures for labeling (elements of) reasoning and argumentation as ‘practical’. Our aim in this endeavor is not only to generate a deeper understanding of the complex relationship between the two fields but also to provide the building blocks for an integrated approach to practical deliberation, which we call the Stakeholder Commitment Approach (SCA). This approach bridges the apparent methodological distance between PR and PA by incorporating insights from both. In particular, SCA yields an extension as well as a refinement of the possible grounds for assigning the label ‘practical’ by looking at the involved stakeholders, the roles assigned to them, and the practical commitments (potentially) generated through the reasoning and argumentation.

We begin the paper, in Sect. 2, by analyzing specific characteristics of principal theories within PR. This analysis includes the relationship between practical and theoretical reasoning, the notion of ‘means-end reasoning’, the nature of the involved premises and conclusion, the distinction between first- and other-person-perspective reasoning, and the distinction between sufficient and necessary means.

Next, in Sect. 3, we first contrast the notions of ‘argumentation’ and ‘reasoning’. Based on an analysis of the literature within PA, we then propose to distinguish between two main approaches: those theories and models taking practical argumentation as publicly performed practical reasoning, to which we refer as the ‘public performance approach’ (PPA), and those based on insights about policy debates, which we call the ‘policy debate approach’ (PDA). Finally, we outline the relationship between these two approaches.

In Sect. 4, we investigate the key discrepancies between the discussed theories in PR and PA and identify the conditions for labeling a particular instance of reasoning or a particular piece of argumentation as ‘practical’. This enables us to locate the source of the discrepancies between the labeling procedures of PR and PA in their understanding and use of commitment.

In Sect. 5, we outline our Stakeholder Commitment Approach (SCA). Based on a systematic variation of the problem-related and communicative roles of the stakeholders involved in practical deliberation, we specify the distribution of commitments invoked. In particular, we propose a distinction between the roles of problem holder and problem solver as a central aspect in identifying argumentation as practical. We then articulate how this novel approach enables the inclusion of second- and third-person practical reasoning as well as a refinement of the models of practical argumentation.

In Sect. 6, we conclude the paper with a short reflection on how SCA integrates insights from the fields of PR and PA, and how it compares to other integrative proposals. Furthermore, we provide an indication of future work.

## Practical Reasoning

Reasoning has been characterized as a cognitive process, a rule-based procedure, a method for belief revision, and a tool for knowledge expansion and decision-making (see, e.g., Walton 1990). It occurs within dialogical settings between conversing interlocutors and within the monological setting of a single reasoner. Furthermore, one is not committed to a cognitive interpretation of reasoning and can likewise think of reasoning as performed by AI agents. The term ‘reasoning’ may also indicate the result of the above activity, e.g., a set of linked statements including premises and conclusions. In this respect, there is a close relationship between reasoning and the study of logic (see, e.g., Streumer 2010).

Practical reasoning, more specifically, can be understood as the type of reasoning invoked by questions about *what to do*. Such questions are referred to as ‘practical problems’, and their answers relate to and motivate the performance of an action, ideally one that satisfactorily addresses the issue at hand. Thus far, in the philosophical literature, the phenomenon of practical reasoning has received considerably more attention than practical argumentation.

In this section, we provide an overview of central themes and challenges in the field of PR. We do this by focusing on those theories that, to the best of our knowledge, have received central attention in the debate.^1^ In particular, we discuss the relation of practical reasoning to theoretical reasoning, its subcategory means-end reasoning, the nature of its premises and conclusion, the distinction between first- and other-person reasoning, and that between sufficient and necessary means. Our discussion serves to facilitate a comparison with our survey of PA in the next section. In Sect. 5, we will provide our own stance on PR.

Present-day debates about what counts as ‘practical’ are informed by Aristotle's distinction between practical and theoretical philosophy. The term ‘practical’ stems from the Ancient Greek *praxis* (i.e., ‘action’) and relates to praxeology, the study of agency and action. It contrasts with ‘theoretical’, stemming from the Ancient Greek *theōría* (i.e., ‘contemplation’ or ‘things looked at’), which refers to knowledge of things, for instance, through perception. Aristotle defined the distinction in terms of *deliberation* ultimately resulting in (rightful) conduct, respectively *contemplation* directed towards attaining truth and knowledge (Hintikka 1991). In light of this classical distinction, as practical beings (i.e., as agents), we engage in practical reasoning when addressing problems of desire, wants, means, and obligation. When engaged in theoretical reasoning, we are considered epistemic beings (i.e., knowers), addressing problems of knowledge, belief, and truth.

While the above distinction may seem straightforward, a complication arises when using it to label the various *elements* of reasoning. For example, assessing a means-end premise of practical reasoning, such as ‘taking the bike is a means for getting to a job interview’, is a theoretical endeavor. The premises of practical reasoning typically concern not only desire, obligation, and intention but also knowledge and belief, reflecting the fact that practical problems engage us in the role of agents as well as knowers. This double role is a distinguishing feature of the practical perspective and is characterized by the agent’s efforts to change the world based on knowing it.

Two other aspects of Aristotle’s account of practical reasoning relevant to understanding present-day debates are his focus on correct (ethical) conduct through deliberation (von Wright 1963**)** and his conceptualization of the conclusion of practical reasoning as an action: “Now when the two premisses are combined, just as in theoretical reasoning the mind is compelled to *affirm* the resulting conclusion, so in the case of practical premises you are forced at once to do it” (*Ethica Nicomachea* 1147a27-28, translation Rackham 1996, our italics). During the twentieth century, a significant shift took place concerning both aspects. First, the study of practical reasoning has been narrowed down to the analysis of means-end inferences. Second, the conclusion of practical reasoning became an intention to act or a normative claim necessitating action rather than the action itself. We now turn to discussing both shifts in more detail.

The focus on means-end inferences stems from the work of Anscombe (2000), who is considered the founder of the modern study of practical reasoning. Means-end inferences are taken to consist of three types of statements: (P1) a premise expressing wants, desire, or motivation; (P2) a theoretical premise concerning the relationship between an action (as a means) and a state-of-affairs (as the action’s outcome); and (C1) a conclusion expressing a normative statement, intention, or action.^2^ Without loss of generality, we focus on wants, necessary means, and normative statements. The corresponding means-end scheme is presented by (S1)

**Table:** 

	(P1) I want *X*;
(S1)	(P2) I know *Y* is the only action leading to *X*;
	(C1) Hence, I must do *Y*.

Von Wright (1963) refers to this scheme as the *primary practical inference*. In (s1) we present an instantiation of this scheme expressing a normative commitment (must) to an action (taking the A-train) that is the only means for accomplishing the given end (to go to Harlem). The example is borrowed from Condoravdi and Lauer (2016).

**Table:** 

	I want to go to Harlem;
(s1)	Taking the A-train is the only way for me to get to Harlem;
	Hence, I must take the A-train.

There are also *secondary* schemes, which replace (P1) with a premise expressing a practical conclusion derived from earlier inferences and, thus, enable chaining.^3^ The reasoning in (s2) is an instantiation of a secondary scheme chained with (s1).

**Table:** 

	I must take the A-train;
(s2)	Only through buying a ticket can I take the A-train;
	Hence, I must buy a ticket.

Means-end reasoning, in short, enables us to determine which actions are required (or sufficient) to secure the want expressed in the first premise. Central to this type of reasoning is the second premise (P2), called ‘the means-end premise’, which typically expresses an instrumentality relation between an action (means) and its outcome (end) and is therefore theoretical in nature.

Means-end reasoning is goal-directed and thus provides immediate guidance in ascertaining ends, desires, and goals.^4^ For this reason, it is considered the most prevalent type of practical reasoning—see, e.g., Clarke (1985) and Walton (2007).^5^ However, the associated scheme (S1) has three features that pose particular challenges. First, since the three statements are of a completely distinct nature (desire (P1), knowledge (P2), and obligation (C1)), the logical status of their relationship is unclear. Second, instantiating (S1) with other perspectives than the above first-person perspective (FPP) causes problems. Third, premise (P2) represents the action as a *necessary* instead of a *sufficient* means to the desired outcome. We address each in turn.

The conclusion of theoretical reasoning is widely recognized to be a belief or a doxastic attitude (cf. Streumer 2010). The nature of the conclusion of practical reasoning, however, is highly controversial. In the literature, we find three main candidates: (i) *action* (Aristotle, *Ethica Nicomachea* and *De motu animalium*—see e.g., Broadie 1991; Dancy 2018); (ii) *intention* (Anscombe 2000; Broome 2001; Lewiński 2021; Raz 1978; von Wright 1963); and (iii) *normative statements* (Audi 1991; Clarke 1985; Walton 2007; von Wright 1972). As seen from the quote at the beginning of this section, Aristotle takes the conclusion of practical reasoning to be the actual performance of an action. Von Wright (1963) argues against this position, taking the conclusion to indicate a setting oneself to act (a perlocutionary effect, nevertheless). Likewise, Anscombe (2000) emphasizes that practical reasoning does not compel any action but instead concludes with intention. According to Broome, concluding intentions is “as practical as reasoning can get” (2001, p. 175). Audi (1991) observes that, although the conclusion (whether it be an intention or a normative statement) is perhaps likely to cause the intended act, causation is not part of a reasoning process. In fact, Searle (2001) argues that the gap between reasoning and deciding is a necessary condition for rationality. This separation of the conclusion of practical reasoning from action serves to explain problematic cases such as failure to act (e.g., through incontinence, change of mind, or intervention) and weakness of the will (*akrasia*)—see Audi (1991) and von Wright (1963). More recently, Dancy (2018) argues for reconsideration and modification of Aristotle’s approach, taking action as the conclusion of PR. The most prevalent approach is to take the conclusion of practical reasoning as a normative judgment: a statement necessitating the agent to certain action; cf. (C1) in scheme (S1).^6^ Whereas intentions and actions do not qualify as propositions implied by a reasoning process, so the argument goes, a normative conclusion does—see Audi (1991) and von Wright (1972). Streumer (2010) adopts the view that all three types of conclusions are possible, representing various kinds of practical reasoning.

The three candidates (i)–(iii) share their consideration of the conclusion as a non-descriptive statement concerning action that differs in nature from the premises from which it is supposed to be derived. Regarding the nature of these premises, Audi (1991) distinguishes between *motivational* and *cognitive* premises. The former motivate the reasoning process through the active desiring and wanting of a certain state of affairs, e.g., (P1) of (S1), and the latter express the reasoner’s beliefs and knowledge about the world and the relations between actions and outcomes, e.g., (P2) of (S1).

Audi stresses that a motivational commitment of the reasoner to the first premise, by means of actively desiring what is stated, and a cognitive commitment to the second premise, by means of actively believing in the accuracy of the means-end relation, are necessary for practical commitment to the conclusion drawn—cf. the sincerity condition of the speech act of asserting (Austin 1962; Searle 1969). Clarke (1985) adopts a similar position, emphasizing that (P1) expresses a *volitional attitude* and (P2) an *epistemic disposition*. He furthermore emphasizes that the wants, desires, or needs described in a volitional attitude additionally require awareness. For Broome (2001), practical reasoning is a rule-based process over cognitive attitudes—including beliefs, desires, and intentions—concluding in intention. Similar positions emphasizing the presence of non-descriptive content can be found in the seminal works of Anscombe (2000) and von Wright (1963).

We emphasize that what these approaches have in common is that they assume the reasoner’s *cognitive* commitment to the content of the reasoning. To avoid confusion, what Audi calls a cognitive commitment with respect to the second premise can be better called an epistemic and doxastic commitment. In what follows, we exclusively use ‘cognitive commitment’ as an overarching term for a reasoner’s volitional, doxastic, epistemic, and normative commitments.

Following the above, the reasoning process captured by the inference (S1) can thus be seen as a transition from motivations and beliefs to a (normative) commitment to action. One of the central challenges concerning such inferences is then to determine the logical relation between the involved statements and the validity of the transition from wants and beliefs to practical necessitation. In almost all of the works mentioned above, we find that the reasoner’s motivational/volitional commitment to the first premise is a distinguishing feature of practical reasoning. It is for this reason that the FPP takes up a central position in the literature on practical reasoning.

This brings us to the next aspect, the essential role of the ‘I’ in models of practical reasoning. As discussed above, the presence of an agent actively endorsing what is desired, wanted, or intended is often considered to lie at the very heart of what makes such reasoning practical. Moreover, the view that practical reasoning must somehow affect the reasoner’s intentions or actions inevitably entails the FPP of PR (Streumer 2010). Consider the following instantiation of (S1) in the third-person perspective (TPP):

**Table:** 

	Billy wants to go to Harlem;
(s3)	Billy knows the A-train is the only means of getting there;
	Hence, Billy must take the A-train.

While the occurrence of ‘must’ in (s3) denotes a normative judgment concerning what is rational for Billy to do, the reasoning itself is often not considered practical, and even labeled theoretical (see the works of Clarke 1985; Hunter 2017; von Wright 1963 for discussions). In (s3), the premises are descriptive (facts and observation) of Billy and are neither (required to be) cognitively nor motivationally endorsed by Billy.^7^ Thus, so the argument goes, (S1) can only be properly practical from the first-person perspective. For von Wright (1963), too, only the first person setting concludes in practical commitment: the rise of an intention. In the third-person perspective in (s3), the conclusion expresses a necessity (normative judgment), which is descriptive as in predictive or reconstructive reasoning. In such settings, the nature of ‘must’ changes from practical (necessitation) to theoretical (rational prediction concerning facts).

Clarke (1985), extensively discusses first- and other-person perspectives of practical inference, distinguishing between second-person perspectives (SPP) and third-person perspectives (TPP). The SPP is employed to *persuade the hearer* to perform the action specified in the inference. The TPP has a different perlocutionary force: it intends to *induce a belief* in the hearer concerning the truth of the conclusion. While von Wright takes the TPP to conclude in a categorical, i.e., detached, normative judgment, Clarke emphasizes that such conclusions are most often hypothetical: ‘*If* Billy wants to go to Harlem, *then* Billy must take the A-train’.^8^ Only when the reasoner themselves cognitively endorses the volitional premise (P1), does the conclusion become categorical. For this reason, the FPP cannot but conclude categorically, since the reasoner always endorses their own wants. Clarke argues that only categorical conclusions are satisfactory for practical reasoning since only those can constitute an appropriate answer to the question ‘What must I do?’ (or ‘What must X do?’). Since the central component of practical reasoning is the endorsement of a want expressed in the volitional attitude, SPP and TPP are commonly considered instances of theoretical reasoning. What von Wright and Clarke (a.o.) have in common, is that they require the reasoner’s volitional commitment in the first premise as a *conditio sine qua non* for identifying it as ‘practical’. A notable exception, in this respect, is Hunter (2017) who argues against the common view that the FPP (and even the SPP) is an essential characteristic of PR.

A final important aspect of present-day models of practical reasoning is the distinction between necessary and sufficient means. The distinction particularly generates certain challenges for the role of choice-making within the reasoning process. Most of the authors mentioned above—except for Clarke (1985), Walton (2007), and Lewiński (2017)—focus on practical inferences based on necessary means only. Hare (1971) points out that the overlooked distinction between *sufficient* and *necessary* means leads to a misleading focus in the philosophy of practical reasoning. Whereas reasoning with necessary conditions is more common to theoretical reasoning (deduction), reasoning with sufficient conditions is more common to practical reasoning (cf. abduction). According to Hare, the problem is that practical necessitation does not follow from sufficient means. The reasoning in (s4) may be considered invalid due to the possibility of alternative sufficient means (e.g., ‘taking the car to Harlem instead of the A-train’) and, thus, does not lead to any conclusion normatively binding the agent to action.

**Table:** 

	I want to go to Harlem;
(s4)	I know that taking the A-train is a way to get there;
	Hence, I must take the A-train.

Hare subsequently observes that, in the case of sufficient-means reasoning, the resolve to act is not a logical conclusion but a *decision* following the reasoning process. Likewise, Clarke (1985) states that decision-making is part of the post-deliberative phase. Walton (2007) similarly distinguishes between practical reasoning as an inferential process and practical deliberation as a goal-directed method of decision-making. Also, recall Searle’s (2001) position that this gap between reasoning and deciding is in fact necessary. In contrast, Lewiński (2017) takes the decision-making process to be at the heart of practical reasoning, incorporating the weighing and selecting of sufficient means into the process.

## Practical Argumentation

Colloquially, ‘reasoning’ and ‘argumentation’ are often taken to be exchangeable notions. When looking at them as products of mental activities, they share many features, most notably their constituents: both a piece of reasoning and a piece of argumentation consist of a structured set of one or more premises and a conclusion. Only in describing these activities, a crucial difference between the two notions becomes visible.

As we elucidated in the previous section, reasoning is an *individual*, *cognitive* process in which an intelligent agent draws a particular conclusion from certain premises. Following the general characterizations and definitions of argumentation provided by, for instance, van Eemeren et al. (2014, pp. 1–7) and Wagemans (2019a, pp. 58–59), we say that arguing is primarily a *social*, *communicative* process in which someone, the ‘arguer’, tries to convince someone else, the ‘addressee’, of the acceptability of a particular conclusion by offering certain premises in support. As the latter is superfluous if the addressee would already accept that conclusion, we can describe the pragmatic aim of argumentation as changing the attitude of the addressee regarding the conclusion from ‘doubt’ to ‘acceptance’.

From a historical point of view, the distinction between reasoning and argumentation is reflected in the coexistence of the philosophical subdisciplines of, on the one hand, logic as the art of reasoning and, on the other hand, dialectic and rhetoric as the art of philosophical debate and that of public speaking, respectively. As emphasized by Aristotle in his debate manual the *Topica*, in contrast to the philosopher, who reasons on their own, the dialectician has to present their reasoning in reference to another party and thus has to consider not only its content but also its arrangement and framing (Wagemans 2019b). In classical rhetoric, the similar tasks of finding (*inventio*), ordering (*dispositio*), and wording (*elocutio*) of the material in preparing a speech for an audience are canonized as the first three of the five so-called ‘tasks of the speaker’. In short, while logic studies the abstract structure of reasoning, either in itself or underlying an argument, dialectical and rhetorical approaches to argumentation study the communicative practice of conducting discussions and giving speeches, respectively (Wagemans 2021).

Based on these considerations, we can see argumentation as a means to invite others to reason. This articulation of the relationship between the two stems from Pinto, who defines ‘inference’ as “the mental act or event in which a person draws a conclusion from premisses” (2001, p. 31) and proposes that “an argument is best viewed as an invitation to inference, that it lays out grounds or bases from which those to whom it is addressed are invited to draw a conclusion” (2001, p. 68). Viewed from this perspective, the relationship between reasoning and argumentation is thus an asymmetric one. Reasoning manifests itself in argumentation without being restricted to it: every argumentation contains reasoning, but not every reasoning is expressed as argumentation.

Practical argumentation, more specifically, can be conceived as inviting an audience to reason about a practical problem. The asymmetric relationship between reasoning and argumentation is reflected in the theories and models developed in the field of PA. While some emphasize the cognitive and inferential aspects of practical argumentation, others focus on the social and communicative aspects. In describing their general characteristics, we propose, therefore, to distinguish between two main approaches.

The first approach focuses on the specific argumentative discourse structure supporting a practical point of view. This approach is close to practical reasoning, using similar terminology for naming the premises involved and taking practical argumentation as externalization or *publicly performed* practical reasoning, to borrow a phrase from Lewiński (2021, p. 435). We shall refer to it as the ‘public performance approach’ or PPA for short.

The second approach uses the notions of ‘policy statement’ and ‘stock issues’ to characterize how debates about practical problems, called ‘policy debates’, are (or should be) conducted. This approach is more remote from practical reasoning as it employs a different terminology and considers any set of premises supporting a practical conclusion as practical argumentation. Since it is inspired by debate theory, we refer to it as the ‘policy debate approach’ or PDA for short. In the remainder of this section, we discuss both approaches in more detail, paying special attention to their usage of the label ‘practical’.

Instead of discussing different variants of the PPA, we discuss here the most recent iteration of the ‘scheme of practical argumentation’ as developed by Lewiński (2015, 2017, 2021). This scheme is based on literature on practical reasoning (a.o., Audi 2006; Broome 2013; Searle 2001) and other instances of the PPA by different authors (a.o., Fairclough and Fairclough 2012; Walton 2007).

Lewiński develops his point of view by criticizing specific aspects of practical reasoning. First of all, as he observes, “[p]hilosophical accounts of practical reasoning […] are still dominated by the first-person perspective of a single reasoning agent” (2021, p. 422)—cf. Section 2 of this article. As a result, the premises and conclusion of such reasoning are named after the propositional attitudes or intentional states involved. Paraphrasing Lewiński (*ibidem*): from my belief that a means *m* leads to achieving a goal *G* and my desire or intention to achieve that goal *G*, it is concluded that I intend to do *m*, i.e., that I should do *m*.

This observation leads Lewiński to propose a first amendment, which draws from the idea of argumentation taking place within a *communicative* setting—see the beginning of this section. According to him, the account of practical reasoning can be improved by considering it as a social activity, thus connecting it to “an argumentative activity of deliberation […]. One main consequence of it is a shift of focus away from the internal propositional attitude of intention to some externalized and collective speech act” (2021, p. 427). In performing this shift, practical reasoning turns into practical argumentation or, as Lewiński puts it: “[practical reasoning], when publicly performed, can better be called practical argumentation” (2021, p. 435).

Another amendment concerns the characterization of the conclusion of practical argumentation. While others have focused on the speech act of ‘imperatives’ and ‘proposals’ as the paradigmatic conclusion of deliberation, Lewiński argues that this is too restricted because it limits the conclusion of deliberation to second person singular and first person plural. He proposes, therefore, to represent the conclusion of practical argumentation as an “action-inducing speech act” (2021, p. 437).^9^

**Fig. 1:**
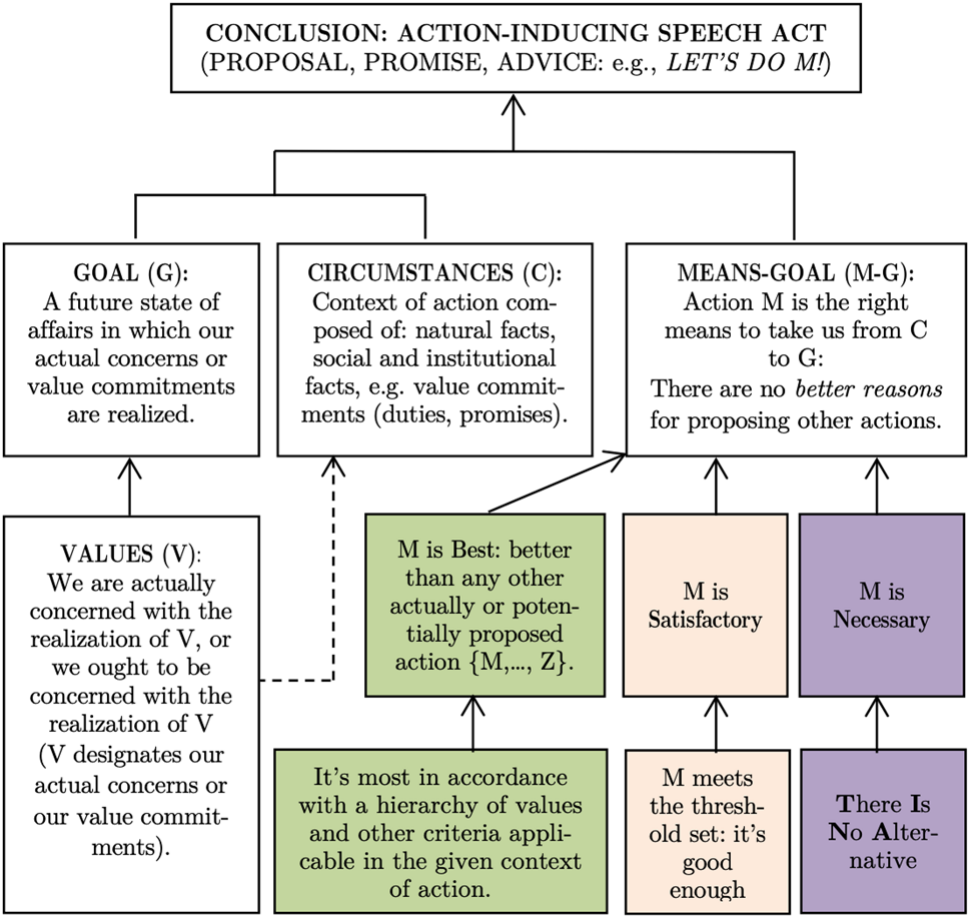
The scheme of PA as presented in Lewiński (2021, p. 436)

As mentioned above, the general scheme of practical argumentation that Lewiński (2021, pp. 435–436) presents is a summarizing account of many sources—in particular, Fairclough and Fairclough (2012)—and includes the two proposals for improvement discussed above. The scheme is pictured in Fig. 1.

While practical reasoning concludes intentional states, the PPA to practical argumentation concludes speech acts from these intentional states. It is in this sense that the PPA takes practical argumentation as *externalized* practical reasoning, i.e., externalized through speech acts.^10^ To see this, observe the similarities between Fig. 1 and the common accounts of PR given in Sect. 2: the central role of means-end premises, the presence of values as desires and normative statements, and the resolution of various sufficient means by means of deliberation. In fact, the PPA developed by Lewiński (2021) is explicitly rooted in the PR theories of Audi (2006), Broome (2013), and Searle (2001).

We believe that the importance of this externalization lies in the fact that through speech acts, more nuances can be observed in the analysis of practical arguments, including the introduction of second-person perspectives (cf. Sect. 2). Moreover, the introduction of speech acts introduces a different type of commitment to the picture: publicly accessible commitments. Furthermore, as Lewiński points out, “[p]ublicity of practical arguments invokes socially and institutionally recognizable commitments” (2021, p. 435). We come back to this in Sect. 4.

The models developed within the second type of approach to practical argumentation, which we have called the ‘policy debate approach’ (PDA), differ in two ways from those philosophical accounts of PR discussed in the previous section. First, they do not typically contain the premises identified in PR, such as desire and means-end premises, but rather contain premises reflecting the arguer’s position regarding the ‘stock issues’ conventionally addressed in a ‘policy debate’. Second, the PDA labels argumentation as ‘practical’ if it is put forward in support of a so-called ‘statement of policy’, which is the central claim supported and attacked by the participants in the debate. Below, we discuss these two differences in more detail, starting with an explanation of the concept of ‘stock issues’.

Stock issues are questions that are typically addressed by the participants in a debate. Their content depends on the domain in which the debate takes place as well as on the nature and content of the debated claim. The notion of ‘stock issue’ derives from that of ‘*stasis*’ or ‘*status*’, a term used in classical rhetoric theory for indicating the main topics or points of discussion in speeches belonging to the judicial genre. The notion has been revived in the twentieth-Century debate tradition, where its application has been extended to other genres of discourse (see, e.g., Braet 1984, 1999; Carter 1988; Freeley and Steinberg 2014; Ihnen Jory 2012; McCroskey and Camp 1964; Schut and Wagemans 2014). In a recent paper, Popa and Wagemans conclude from a survey of relevant literature that descriptions of stock issues usually contain one or more of the following points:

(i) Stock issues are general in the sense that they apply to more than one interaction and often, by definition, to all discussions of a certain type. For example, in a legal discussion about guilt, arguers usually draw upon the deeds of the ones involved, their knowledge of the risks, aggravating and attenuating circumstances, alibis, and the like. […]
(ii) Stock issues have normative force in the sense that the speakers are expected to address them in their argumentative discussions—choosing and ordering them relative to the institutional setting in which the discussion takes place […].
(iii) Depending on the context, stock issues are accompanied by a *decision rule* which stipulates the weight of each issue in the ultimate decision and thus directs the parties from exchanging arguments pro and con to taking a decision based on the exchange. In the legal context, such decision rules are stipulated by law […]. In less formalized contexts, more often than not they remain implicit and thus need to be reconstructed in order to fully understand the motivation for the decision. (Popa and Wagemans 2021, p. 130)

While each genre of argumentative discourse has its own specific set of stock issues, in general, the term is reserved for the standard issues to be addressed in so-called ‘policy debates’, which center around a particular statement of policy (e.g., ‘The government should increase income tax’). The first of these issues is called ‘problem’ (or ‘harm’), and the main reason the proponent should address this issue is that when there is no problem, there is no need for action either. Even if the statement of policy defended by the proponent contains the best plan among competing alternatives, if the *status quo* is unproblematic, there is no need to change it.

A similar reason applies to the two stock issues called ‘urgency’ and ‘inherency’. Apart from the existence of a problem, the proponent should prove that the problem is urgent and inherent to the *status quo*, i.e., caused by a factor that is characteristic of or belongs to the present situation. Even if a problem has been identified, if it is neither urgent nor inherent in the *status quo*, there is no need for action.

A fourth stock issue is called ‘solvency’. This issue relates to the requirement that the policy should have an effect such that the problem is solved. Another stock issue, called ‘workability’, requires the policy to be feasible. The last point that the proponent must demonstrate is that, in case of undesirable side effects, the positive effects outweigh the negative ones. This stock issue is called ‘advantages’ or ‘cost–benefit’.

Within an actual policy debate, the specific content of the premises put forward in support of a statement of policy may seem somewhat arbitrary since they depend on the particularities of the case at hand and the subjective contributions of the participants to the discussion. The general idea behind the PDA is that these premises reflect, to a lesser or greater extent, the stock issues involved. The latter express particular presumptions, expectations, and conventions regarding how such debates are usually conducted and therefore have a certain normative force.

**Fig. 2:**
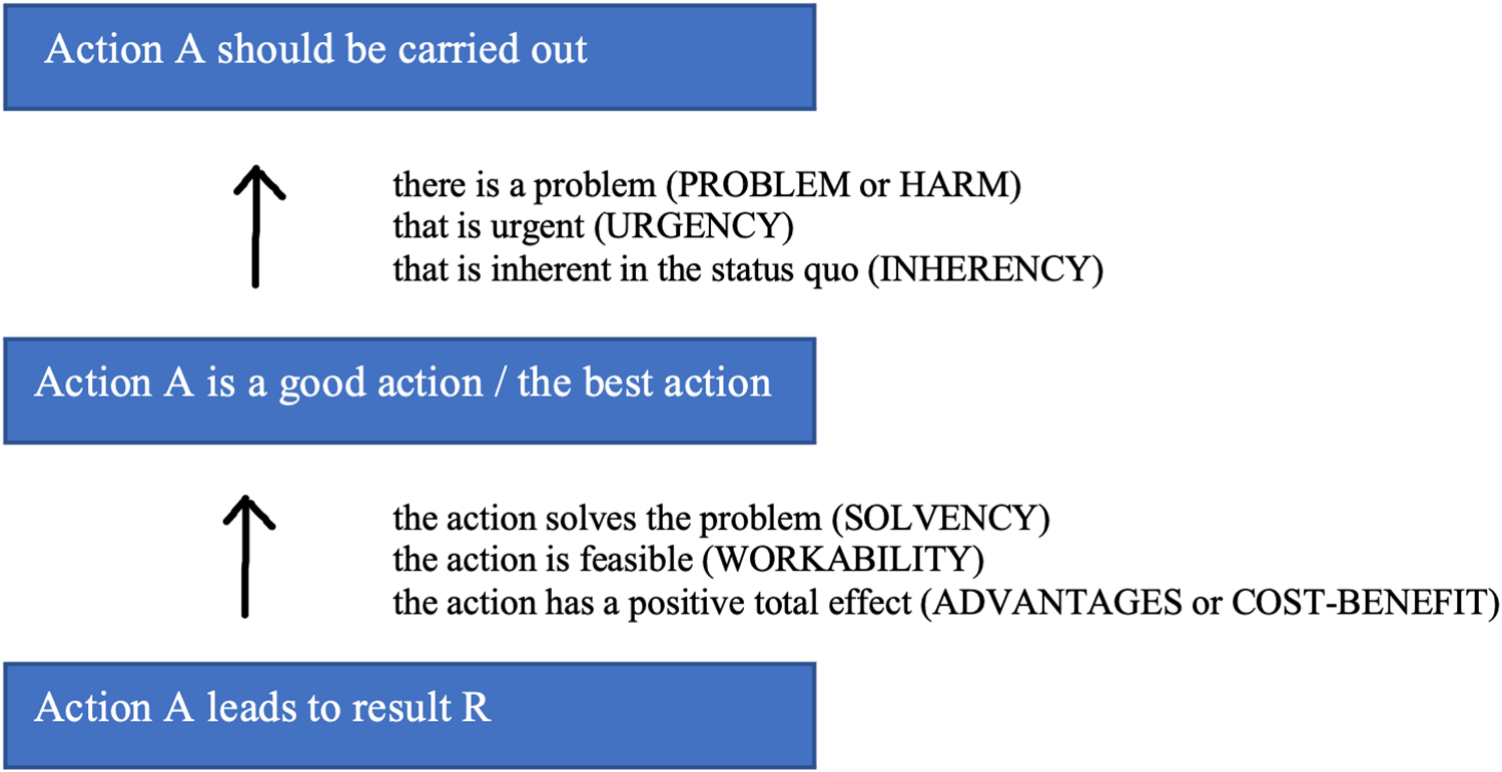
A generic argumentation structure for policy debates

In Fig. 2, we picture the stock issues described above in the form of premises, i.e., in the way they are addressed by the proponent in the debate. The stock issues ‘problem’ or ‘harm’, ‘urgency’, and ‘inherency’ address potential criticisms regarding the relationship between the premise that the proponent is defending a good action, (or when there are alternatives, the best action) and the conclusion that the action should be carried out. This group of stock issues can therefore be interpreted as premises supporting the relationship between the conclusion and the second premise. The stock issues ‘solvency’, ‘workability’, and ‘advantages’ or ‘cost–benefit’ address potential criticisms regarding the relationship between the second premise that the proposed action leads to the result in question and the first premise that the proposed action is good or the best. This second group of stock issues can, therefore, be interpreted as premises supporting the relationship between the second and the first premise.

This representation of the main stock issues in a generic argumentation structure for policy debates is modeled on Wagemans (2016), who indicates how the issues specified in classical rhetorical status theory can be interpreted in terms of a generic argumentation structure for legal debates. The primary purpose of the representation is to indicate the argumentative function of the stock issues in policy debates. In the literature, one finds several partial instantiations of this generic structure. Ihnen Jory (2012), for instance, provides a detailed representation of stock issues as supporting premises in pragmatic argumentation, and van der Geest (2015) presents a structure of argumentation supporting a choice containing several stock issues as sub-premises.

While the PPA and the PDA thus differ in their characterization of the content and structure of the premises of practical argumentation, their conceptualization of its conclusion is very similar. As is clear from the general model just presented, the PDA takes practical argumentation as support for *statements of policy*. This is related to its origins in debate theory, where it is common to make a distinction between three types of statements participants in a debate may put forward (see, e.g., Broda-Bahm et al. 2004; Kruger 1975; Schut and Wagemans 2014; Skorupski 2010; Freeley and Steinberg 2014; Wagemans 2023): statements of fact, statements of value, and statements of policy. Wagemans (2023, p. 125) defines a statement of policy as “a directive or hortative statement that expresses advice to do something or to refrain from doing something”. Statements of policy typically predicate of a specific act (course of action, policy) that it should be carried out and may also include as their constituents an actor, an object of the act, and a temporal indication. An example of all these constituents being present is ‘The city of Vienna should legalize soft drugs in 2023’. Linguistically, statements of policy are expressed in various ways: as incitements, advice, imperatives, or proposals (*ibidem*). In this respect, the PDA conceptualization of the conclusion is close to that of the PPA, which works with a similar set of statements expressed in terms of speech act theory.

Last, we briefly indicate the relation between the PDA and PPA, and their distance to PR. As is clear from the above analyses, the PPA takes practical argumentation as publicly performed practical reasoning employing a variety of speech acts. The PDA, by contrast, takes practical argumentation as any kind of argumentation with a statement of policy as its conclusion, irrespective of its premises. By implication, every instance of a practical argument in the PPA is also an instance in the PDA (or can be rephrased in terms of it), but not vice versa.

Concerning PR, we find that the PPA is closer in many respects, assuming the presence of practical reasoning in any practical argument. However, an important difference is that the PPA includes second- and third-person perspectives, mainly by virtue of the speech act involved. The PDA is more remote from PR since it considers particular instances of arguments that are by definition excluded in the main approaches to PR as ‘practical’. We now turn to analyzing these discrepancies in greater detail.

## Understanding the Discrepancies

In Sect. 3, we characterized the relationship between the social, communicative activity of argumentation and the individual, cognitive activity of reasoning by describing argumentation as an invitation to engage in reasoning. The arguer invites, so to speak, the addressee to reconstruct a particular instance of reasoning by offering a set of premises and a conclusion as its ‘materials’. Reasoning thus occurs within argumentation, providing the arguer with the content to be communicated and the addressee with the means for reconstructing the reasons offered. The arguer’s aim in this activity is to incite commitment to the conclusion on the part of the addressee.

Argumentation also brings along specific commitments related to the act of arguing itself. As van Eemeren et al. put it:

rather than being just an expressive act free of any obligations, as a rational activity of reason, argumentation involves putting forward a constellation of propositions *the arguer can be held accountable for*. The commitments created by argumentation depend not only on the propositions that are advanced but also on the communicative function they have in the discourse. (van Eemeren et al. 2014, p. 5, original italics)

To understand the discrepancies between PR and PA (both PPA and PDA), it is helpful to note they have a different understanding of commitment: in PR, commitments are actual commitments in terms of cognitive attitudes, and in PA, commitments are reasonably attributable commitments based on the felicity conditions associated with the type of utterance, i.e., the speech act involved. While, in the context argumentation, the arguer may try to elicit cognitive commitments by inviting the addressee to reason, attributable commitments can be seen as public or interpersonal commitments, i.e., they derive from argumentation as a form of communication governed by social conventions. In the remainder, we recapitulate how the discussed approaches assign the label ‘practical’ and how this relates to the distribution of various types of commitments among the participants in the activities of reasoning and arguing.

As discussed in Sect. 2, most accounts of PR only consider reasoning from the first-person perspective (FPP), concluding in ‘I should (not) do X’, as practical, and label any reasoning with a conclusion addressing an agent other than the reasoner as ‘theoretical’. The practical necessitation expressed in the conclusion represents the agent’s normative or intentional commitment to a given action. For assigning the label ‘practical’, however, the reasoner must additionally have specific cognitive commitments to all the involved premises, containing at least one volitional or normative commitment. The reasoning in (1) below, for instance, generates a practical commitment to the prescribed action only if the reasoner endorses both the involved desire (a volitional attitude) and the belief concerning the best means available (an epistemic attitude).

(1) I want to go to Amsterdam, and taking the train is, all things considered, the most optimal, hence I must take the train.

Thus, we say that in PR it is the notion of commitment as *cognitive attitude* together with the expression of an intention or necessitation in the conclusion that justifies the label ‘practical’.

The PPA, as we explained in Sect. 3, opens the door to labeling reasoning from the second-person perspective (SPP) and the third-person perspective (TPP) as ‘practical’ because it generalizes practical reasoning to practical argumentation via the inclusion of speech acts. That is, practical reasoning may conclude in practical argumentation by speech acts such as ‘Just take the bike!’. At the same time, we point out that the PPA assumes a more general notion of practical reasoning than the common approach to PR. Take, for instance, the practical argument (2a)–(2b):

(2a) You want to be at work as soon as possible, so you should take the bike.

(2b) Just take the bike!

The reasoning in (2a) is considered theoretical reasoning, not practical, in common approaches to PR, even though (2a) and (2b) together classify as a practical argument in the PPA. Therefore, we argue that the PPA assumes a wider conception of practical reasoning, closer to the inclusive account provided by Clarke (1985). Furthermore, the use of ‘practical’ in the PPA is closer to PR than the one in the PDA, for which all argumentation in support of statements of policy is labeled as ‘practical’. We emphasize that the PPA’s account of commitment differs from the one adopted in PR. Rather than referring to cognitive attitudes—i.e., in psychological terms—the PPA conceives commitments as *reasonably attributable commitments* following from the felicity conditions associated with performing specific speech acts, i.e., in communicative terms (see also Macagno and Walton 2018).

In the PDA, the nature of the claim receiving support through argumentation is the *only* criterion for such labeling, which occurs irrespective of whether the support contains, for instance, premises expressing volitional attitudes. In contrast to practical reasoning, despite the central role of commitments in argumentation theory in general (see, e.g., Walton and Krabbe 1995), the notion of commitment does not play a decisive role in the PDA understanding of ‘practical’. For example, when (1) occurs in an argumentative setting, it would be classified as practical argumentation by virtue of the nature of its conclusion only.

In both the PPA and the PDA, practical argumentation involves types of reasoning that are commonly not labeled ‘practical’ in the context of practical reasoning.

Thus, the label ‘practical’ is assigned differently for PR and PA (PPA and PDA). Since any argumentation contains reasoning, an instance of reasoning/argumentation, such as for example (3), will be labeled differently depending on the perspective from which it is analyzed.

(3) You should take the bicycle since it is the best means of transportation in the center of Amsterdam (given that you want to move around in Amsterdam).

When conceived as reasoning proper, the general approach in PR is to label (3) theoretical. That is, the conclusion does express necessity, but only in a descriptive way: *my* reasoning cannot incite a commitment or disposition to act in *you*, the subject of the conclusion. When analyzed in the context of argumentation, by contrast, (3) is considered practical by virtue of the nature of the conclusion ‘You should take the bicycle’, which is an imperative in the PPA and a policy statement in the PDA.

How to explain this discrepant use of ‘practical’? To answer this question, we first note that the labeling procedures in PR and PA adopt two different perspectives on the reasoning/argumentation under scrutiny: both assign a central role to the nature of the involved claim, be it a proposition or a speech act. Still, within PR, the label ‘practical’ is assigned under stricter conditions, namely, that the cognitive commitments of the reasoner must be of a specific kind (volitional, intentional, or normative). These stricter conditions are why PR focuses mainly on FPP reasoning, excluding SPP and TPP altogether, and may thus be identified as the cause for criticism of PR from the perspective of PPA (cf. Lewiński 2021). From the perspective of PA, we see that, although argumentation might generate various cognitive and attributable commitments, these commitments do not play a decisive role in calling instances of argumentation ‘practical’, neither in the PPA nor the PDA. For the PDA this is straightforward. To see this point for the PPA, we observe that the types of premises and the speech acts involved qualify a piece of argumentation as practical. The use of speech acts inevitably involves commitments, but it is the speech act that serves as a classifier.

From this analysis we conclude that the notion of ‘commitment’ plays a central role in understanding the discrepancies between the common procedures in PR and PA for labeling something as ‘practical’. In the next section, we use this insight as a starting point for developing an integrated approach to practical deliberation that specifies the distribution of commitments among the stakeholders involved in the process.

## The Stakeholder Commitment Approach

Based on our analyses of the relationship between theories and models of PR and PA, we propose in this section our Stakeholder Commitment Approach (SCA). As the name indicates, this approach centers around the commitments of the stakeholders engaged in practical deliberation, be it reasoning or argumentation. Starting from the idea that an interactive engagement to convince the addressee to accept, and so commit to, a practical conclusion is a central aspect of practical argumentation, in our approach to practical reasoning and argumentation, we focus on the different roles of stakeholders and the way this influences the distribution of commitments among them.

Before explaining the SCA in full detail, we take three preparatory steps. The first one is to introduce a distinction between the *problem holder*, i.e., the party that holds the problem, and the *problem solver*, i.e., the party that is invited or necessitated to solve it. For most accounts of PR, we note, this distinction collapses because, in the FPP, the problem holder is the problem solver (an exception is Clarke 1985).^11^ The one who reasons is both the one who desires and the one who is necessitated (or, more generally, addressed) by the conclusion, so commitment relates to a single agent called the reasoner. The SCA employs this distinction between problem holder and problem solver, together with insights from PA, to render accounts of PR more inclusive, e.g., not prima facie excluding SPP and TPP.

In PA, both in the PPA and PDA, the situation is different as the roles of problem holder and problem solver may be distributed over the (in)directly involved parties (e.g., *I, you, we, they*) in many ways. Consequently, commitments can likewise be distributed among different parties involved. While this distribution is underspecified in PA, the SCA provides a fine-grained specification of parties and commitments.

As a second preparatory step, we adopt the notion of ‘practical commitment’ as an additional criterion for labeling argumentation as ‘practical’. Henceforth, using the terminology of step one, we define a practical commitment as a commitment to action by the *problem solver*. Consider (4), in which the identified problem is ‘being on time at a band rehearsal’. The problem holder is me. The problem solver is you. Namely, by lending me your bike, I can be on time for my rehearsal.

(4) To make it in time for my band rehearsal, you must lend me your bike.

We say there is a practical commitment in (4) when you commit as a problem solver to lending me your bike. For instance, your replying with ‘of course, here is my bike’ would yield such a practical commitment. We use the term practical commitment to emphasize the commitment’s relation to action and to differentiate it from theoretical commitments such as doxastic commitments (‘I believe that…’) and epistemic commitments (‘I know that…’). Nevertheless, the two do not form an exhaustive partition, e.g., neither subsume bouletic commitments (‘I want…’).

The previous sections demonstrated that reasoning and argumentation involve different types of commitment: reasoning deals with cognitive (or psychological) commitments, whereas argumentation deals with commitments that can be reasonably attributed based on communicative conventions governing speech acts. Combined with the distinction between practical and theoretical commitments, we have at least four types of commitments. In brief, with the SCA we obtain different degrees of ‘practical’, based on how various types of commitments are distributed among the arguer, the addressee, and third parties, as well as among the problem holder and problem solver.

As a third and final preparatory step, we emphasize that arguing *aims at generating* cognitive commitments and thus may cause reasoning previously labeled as ‘theoretical’ to become ‘practical’. Consider (5), put forward by me in a dialogue between you and I.

(5) Remember that we want to go to Amsterdam, and since this is only possible if you fill out this form in time, you should fill out the form! (It appears that I am slightly stressed.)

The problem solver in (5) is you and the alleged problem holders are you and I together. However, whether (5) is practical depends on the context of this dialogue, e.g., see (6) in response to (5). From the perspective of PR, (5) is theoretical, and although I have a volitional commitment to the motivational premise (wanting to go to Amsterdam), the conclusion is not practically necessitating. However, in arguing with you, I *invite* you to reconstruct my reasoning and to accept the corresponding conclusion. In doing so, I invite you to reconstruct my theoretical reasoning *practically*. Depending on whether you (i) accept my reasoning, (ii) are volitionally committed to the first premise, and (iii) are epistemically committed to the second, the reconstructed reasoning will become practical. If you disagree with either one of (i)–(iii), you may still hypothetically agree with the reasoning. For instance, you may retort with (6).

(6) Indeed, *if* we want to go to Amsterdam, I must indeed fill in the form (but I don’t want to).

In your response, I may attribute to you a theoretical commitment, not a practical one.^12^ Only if the addressee is committed to the involved premises in the accepted argument, and one of those commitments is practical or volitional, does the reconstructed *reasoning* become practical. Recall that the PPA and the PDA would label all of (1)–(6) practical.

We stress that although the activity of arguing aims at generating cognitive commitments, the assessment of such commitments is done via communication. That is, the arguer (or audience, for that matter) can only evaluate the success of an argument through the communicated response of the addressee and the attributable commitments it generates. This differentiation is rather intricate but should not cause any complications in what follows. Namely, henceforth, when we talk about commitments in a piece of argumentative discourse, we mean attributable commitment and assume that the attribution is the result of some (implicit) communication.

The SCA systematically investigates practical argumentation by looking at the stakeholders, the roles assigned to them, and the practical commitments (potentially) generated through the reasoning contained in the argumentation. Stakeholders are the actual persons (indirectly) involved in the argumentation, and they can be assigned (several) different roles. In our approach, we distinguish between *problem-related* roles—problem holder and problem solver—and *communicative* roles—arguer, addressee, and third party. The communicative role of ‘third party’ is assigned to those stakeholders absent in the activity of arguing but present in the subject of the argumentation. For example, the argumentation in (7) has three stakeholders: ‘I’, ‘you’, and ‘the government’. You and I have the role of problem holder, whereas the government has the role of problem solver. The communicative roles of I, you, and the government are arguer, addressee, and third party, respectively.

(7) You and I want climate change to stop, so the government should reserve more of its annual budget for reducing CO_2_ emissions.

Table 1 represents the different distributions of roles for a given instance of argumentation, which enable us to determine the presence of practical commitments. For instance, when the arguer is the problem solver, there is practical commitment. Whenever the addressee is the claimed problem solver, there is an invitation to a practical commitment by means of argumentation. When neither the arguer nor the addressee is the problem holder or solver, there is no potential for practical commitment because none of the stakeholders has a problem-related role. The different distributions of *presence of, invitation to,* and *absence of* practical commitment provide us with a way to distinguish between different degrees of practicality in argumentation. Instance (I) is the most practical since there is practical commitment concerning both the problem and its solution. In the case of (IV), there is an invitation of practical commitment for the addressee. In (VI), only the solution can potentially be practically committed to. Case (IX) is the least practical since, although we can identify a problem holder and solver (cf. statements of policy), due to the third party’s absence, there is no potential of generating practical commitment through this instance of argumentation.^13^

**Table 1:** Perspectives, roles, and practical commitment in the Stakeholder Commitment Approach (SCA)

Case	Perspective	Problem holder	Problem solver	Neither	Practical commitment of the problem solver
I	FPP	Arguer	Arguer	Third party	Present
II	SPP	Arguer	Addressee	Third party	Invited
III	FPP	Addressee	Arguer	Third party	Present
IV	SPP	Addressee	Addressee	Third party	Invited
V	FPP	Third party	Arguer	Addressee	Present
VI	SPP	Third party	Addressee	Arguer	Invited
VII	TPP	Arguer	Third party	Addressee	Absent
VIII	TPP	Addressee	Third party	Arguer	Absent
IX	TPP	Third party	Third party	Arguer/addressee	Absent

In its most general characterization, we may say that SCA stipulates that an instance of argumentation is practical (to some degree) whenever there is a problem holder and problem solver identifiable in the argumentation. The degree of practicality is determined by the different commitments distributed among the stakeholders. We point out that within the context of PA, the cognitive commitments of the parties involved are not and are also not required to be considered because in argumentation, attributable commitments suffice (see Sect. 4 and the beginning of this section). Last, we remark that in the case of group deliberation, we take ‘we’ as the collective arguer consisting of both the arguer and the addressee.

The distribution of roles in (I) tells us of the presence of practical reasoning in the arguer, who is both the problem holder and the problem solver. The argument thus communicates a commitment of the arguer to both the problem (cf. volitional commitment) and its solution (cf. normative commitment). The reasoning in (8) is an instance of (I). In communicating the argument, the arguer presupposes some doubt in the addressee. For instance, I believe that you disagree that I should take the bike because you might think public transport is faster. In that case, you disagree theoretically with my practical reasoning.

(8) If I want to be at work on time, I should take the bike since it is the fastest option.

In (II), the arguer tries to convince the addressee to solve their problem for them. Part of the argumentation here is directed at convincing the addressee that the arguer’s problem must be solved. Take, for instance, (9), where you are my parent.

(9) You must make me a sandwich since I am hungry.

You may agree, in (9), with me being hungry, but still, you may believe that you are not the one that must solve the problem. Your response may be the following: ‘Well, you are old enough to make your own sandwich’.

Instance (III) can be seen as an argument that aims at helping the addressee with solving their problem. In such cases, to qualify as an argument, the arguer may, for instance, doubt whether the arguer is the right person to solve the problem, or whether the addressee has a problem at all. The reasoning in (10) is an example. In those cases, the practical commitment on behalf of the arguer concerning solving the problem indicates that the problem solver has an implicit commitment to the addressee’s problem (e.g., ‘I don’t want you to be hungry’ or ‘I am your parent and I have a duty to make sure you are not hungry’).

(10) You are so hungry! I should make you a sandwich.

Scheme (IV) can be seen as a form of advice: ‘If this is your problem, you should do that’ (or it may be conceived of as patronizing ‘you have this problem, you should do that to solve it’). In order to qualify as an argumentation, and not as an explanation, we must assume here that the addressee who receives advice for their problem, will not readily accept this advice: you may disagree with having a problem (that needs to be solved), with the proposed means to solve it, or with some of the inferences applied.

The instances (V) and (VI) of Table 1 are practical in the sense that the arguer (V) or addressee (VI) may become practically committed to a certain action based on the argumentation. It must be noted that although the problem holder is the third party, once the arguer or addressee becomes practically committed to solving the problem, it is reasonable to assume a commitment from the solver to the problem itself (which is not only hypothetical). In such cases, there could be an additional motivational premise at play to ensure the practical commitment to solving the problem (e.g., I want to help solve the third party’s problem). (11) is an example of (V).

(11) They want to go to Amsterdam, so I must get the paperwork ready.

Communicating an ‘I must’, such as in (11), suggests a commitment to the problem that has to be solved (e.g., ‘I want them to go to Amsterdam’). Example (12) is an instance of (VI). In such cases, there is no initial practical commitment involved, although the addressee is invited to solve the third party’s problem. Whether such commitments, in fact, arise is something that the course of the discussion will decide.

(12) They want to go to Amsterdam, so you must get the paperwork ready. (I might be your boss, in a grumpy mood.)

Both cases (VII) and (VIII) can be called practical argumentation due to the fact that either the arguer or the addressee is committed to the problem being solved. The fact that the problem solver is a third party indicates that no direct practical commitments can be generated through the argumentation. Think of cases in which the addressee and the arguer are trying to find out (theoretically) what the best conduct of the third party would be in order to solve the arguer’s problem, as in (13), or the addressee’s.

(13) I want climate change to stop, so the government should reserve more of its annual budget for reducing CO_2_ emissions.

In the case of (IX), where both the problem holder and problem solver are third parties, the argumentation occurring between the arguer and addressee is not about generating practical commitments anymore. Such argumentation may be rightly called theoretical since none of the commitments involved is directed toward action. The argumentation in (14) is an instance of such third-party argumentation. We stress that we label such argumentation still as practical due to the presence of an explicit problem holder and problem solver.

(14) Billy wants to go to Harlem, so Eduard should book Billy’s ticket for the A-train today. (Provided the arguer and addressee are neither Billy nor Eduard.)

We point out that under the PDA all instances (I)-(IX) would be labeled practical with the same degree. We can now better understand how the SCA provides refinements in types of practical argumentation by looking at problem-related and communicative roles in the argumentation.

By taking into account the different roles of the involved stakeholders, we can also introduce an extension of the analysis of practical reasoning. Whereas traditional approaches often take the distribution in (I) of Table 1 as the only case of practical reasoning proper, we can now include SPP reasoning into the analysis: in case of successful communication on behalf of the arguer, the addressee accepts the invitation to reasoning and commits practically to both the problem and its solution. In this case, the arguer contributes to *generating* practical reasoning on the side of the addressee, namely, as a reconstructed instance of theoretical reasoning on the side of the arguer.

Instances (I)–(IV) of Table 1 are practical in the sense that all relevant commitments are distributed over the directly present parties: arguer and addressee. Instance (I) would be a case of FPP practical reasoning in an argumentative context, (IV) denotes a case of SPP practical reasoning. (According to Clarke 1985, those cases would be practical.) Furthermore, the role assignments in (II) and (III) are instances of reasoning which have not yet been properly investigated in the context of practical reasoning. For example, here, one may investigate whether the reconstructed reasoning on the side of the addressee is practical whenever the addressee commits to solving the problem to which the arguer is committed. What does this say about the commitments of the addressee? Does an epistemic commitment suffice, e.g., ‘I know that the arguer has problem X’? Or is there a sub-problem expressed through the volitional commitment, e.g., ‘I want to solve the problem of the arguer’? In the latter case, there is practical reasoning on the side of the addressee. In that sense, the communicative model presented in the SCA tells us something about the (potential) presence of practical reasoning in the arguer and addressee. As an example, consider the argumentation in (15).

(15) I want to go to Amsterdam for work, so Billy must sign my travel forms (Billy is from human resources).

Following von Wright (1972), reasoning instances such as the one in (15) implicitly contain the potential of practical reasoning. Namely, each claim necessitating an action to a second or third party that is not the problem holder implies a necessitating conclusion for the problem holder themselves. This is expressed in (16), which can be seen as a practical reasoning consequence of (15).

(16) I want to go to Amsterdam for work, so *I must* ensure that Billy signs my travel forms.

Concerning the SCA, this means that any of the instances (II) and (VII) imply arguments of the form (I), and instances of (III) and (VIII) imply arguments of the form (IV). For instance, (15) is an instance of (VII), which can be rephrased as the practically committed argument (16) belonging to category (I).

Hence, through the SCA, what is commonly taken as theoretical reasoning is susceptible to reconsideration. A reconceptualization would be more in line with the inclusive account provided by Clarke (1985). Additional to classifying second- and third-person practical inferences as instances of practical reasoning, one may inquire about the distinction between these two perspectives and the role of communication, absent in the TPP, but often present in SPP through an invitation to reproduce and accept the offered reasoning. In fact, the SPP accommodates a starting point for practical argumentation. In common accounts of practical reasoning, such nuances between SPP and TPP are lost. By putting aside SPP and TPP as both theoretical and thereby moving them outside the scope of practical reasoning, one a priori excludes notions fruitful to a better understanding of ‘practical’.

## Conclusion

In this paper, we analyzed how prominent theories in the fields of Practical Reasoning (PR) and Practical Argumentation (PA) employ the label ‘practical’. After explaining the discrepant use of this label, we developed an integrated approach to practical deliberation called the Stakeholder Commitment Approach (SCA). This approach yields an extension as well as a refinement of the possible grounds for assigning the label ‘practical’ to instances of reasoning and argumentation by specifying the distribution of the various types of commitments among the stakeholders. Both the extension and the refinement are based on our introduction of a distinction between the roles of problem holder and problem solver. While the extension serves to include those aspects of reasoning that influence practical reasoning but are commonly prima facie excluded, the refinement serves to distinguish aspects of reasoning and argumentation that are often grouped together.

The extension is accomplished in comparison to PR. A central feature of the theories and models developed within this field is the plurality of constituents involved in the reasoning process: practical reasoning contains (1) a premise expressing the agent’s motivational disposition towards the problem to be solved via an actual desire, want, or intention, i.e., a volitional attitude (in the case of secondary inferences, such motivational disposition is at least indirectly present); (2) theoretical reasoning by virtue of means-end premises expressing the reasoner’s epistemic dispositions on actions and their potential outcomes; (3) a non-descriptive conclusion often expressing a normative judgment or intention. Since the reasoner must be cognitively committed to the premises for the conclusions to be practically binding, practical reasoning is commonly considered as reasoning from the first-person perspective. Compared to PR, the SCA is more inclusive in that it considers not only first- but also second- and third-person reasoning as practical. The differentiation between these perspectives takes place by looking at the assigned roles of the stakeholders.

The refinement is achieved in comparison to PA. Based on an analysis of prominent theories and models developed within this field, we introduced a distinction between the ‘public performance approach’ (PPA) and the ‘policy debate approach’ (PDA). Compared to PR, in both these approaches, the notion of commitment plays a less important role in classifying argumentation as practical. Their criteria for labeling argumentation as ‘practical’ are not formulated in terms of the commitments invoked but in terms of the nature of the conclusion (the speech acts expressing it and statements of policy, respectively). Nevertheless, in the PPA, commitments are an immediate and important consequence of the involved speech acts. The SCA, in contrast, investigates practical argumentation by looking at the stakeholders, the problem-related and communicative roles assigned to them in the argumentation, and the commitments (potentially) attributable through the argumentation. As a result, it facilitates the characterization of a larger variety of different instances of practical reasoning and practical argumentation.

We stress that our approach is fully compatible with both the PPA and the PDA. Through the SCA, we gain a better insight into the practical aspects of argumentation by looking at how the distribution of certain roles influences the practical commitments (potentially) generated through acts of communication. Furthermore, the SCA allows for a better understanding of the relationship between practical reasoning and practical argumentation. On our account, practical argumentation is both more than a communicative externalization of PR (cf. PPA) and more than the mere presence of policy statements (cf. PDA).

Among the remaining challenges, we count the delineation of attributable commitments in group deliberations, where the ‘we’ seems to include both the arguer and the addressee. One may wonder, for instance, whether ‘We want to go to the party’ involves a practical commitment to the problem of all members of ‘we’ or only of the speaker. Another issue arises when the problem or the problem holder remains implicit, as is the case in, for example, ‘It starts raining, you should hurry home!’ We leave such investigations for future work.

Furthermore, it would be interesting to study the relationship between the criteria for labeling a piece of argumentation ‘practical’ and those for assessing the success of the argumentation. Successful communication may lead to a practical commitment. For instance, when I argue that you should be on time for our meeting, and you agree. However, it is not an exhaustive criterion, and failed argumentation may be practical too. For instance, you argue that I should do my taxes today, and I disagree, saying that the deadline is only next week, but I will do my taxes tomorrow instead. The initial argumentation may not be successful, but the overall argumentation remains practical.

Apart from addressing these challenges, further research needs to be carried out to compare the SCA to other theories and models proposing or assuming a more integrative approach. One example of such an approach is Sàágua and Baumtrog’s (2018) ideal model for practical reasoning and argumentation, which provides a detailed specification of argumentation schemes and allows for analyzing the commitments involved from the perspective of argumentation and reasoning. Another example is Baumtrog’s (2018) multifaceted differentiation between dialectic, dialogue, and quasi-dialogue, reasoning and argumentation, among individual and multiple participants, which challenges the assumption that argumentation always involves conversational interchange with one (or more) other person(s) or imagining such an interchange. On a more general level, it would also be interesting to explore how SCA’s viewpoint on the relationship between reasoning and argumentation relates to recent developments in cognitive psychology, which suggest empirical test results can be better explained if we hypothesize that the function of reasoning is argumentative rather than corrective (Mercier and Sperber 2011).

With our new approach to practical reasoning and argumentation, we hope to contribute to connecting the two fields and exchanging insights between them, importing additional nuances in the investigation of what is ‘practical’. We are better positioned to apply such a conceptual modification if we understand why the label is used in distinctive ways. The extensions and refinements of the conceptual framework not only facilitate clarity but may also yield novel questions and investigations within PR and PA and where they interact.

## Data Availability

Data sharing is not applicable to this article as no new data were created or analyzed in this study.

## Footnotes

1 We refer to the work of Streumer (2010) for an overview of various other approaches to and challenges of PR. See Kauppinen (2018) for a division of approaches to PR into those that are rule-based, which take PR to be about attaining intentions, and those that are reasons-based, which take PR to be about agents responding correctly to reasons.

2 For Anscombe (2000), ‘wants’ function as instigators of practical inference but are, strictly speaking, not part of the inference. Instead, wants give rise to the first premise concerning a judgment of value expressing that the agent considers what it wants to be good.

3 Clarke (1985), von Wright (1963), and Walton (2007) all talk about chaining inference schemes.

4 This view strongly relates to the influential Belief-Desire-Intention model developed by Bratman (1987) and has been extensively investigated in other fields such as Artificial Intelligence (Rao and Georgeff 1995).

5 Notwithstanding the focus on means-end inferences, many philosophers stress that this inference is just one instance of a variety of practical reasoning schemes. Audi (1991) emphasizes the plurality of practical reasoning, of which he argues that means-end reasoning is a common practical scheme. Clarke (1985) takes practical reasoning to comprise a variety of inference schemes, ranking the means-end scheme among one of them. Other authors mentioning the plurality of practical schemes are Broome (2001) and Walton (2007). Walton introduces a distinction between a narrow conception of practical reasoning, which is means-end reasoning, and a broader conception which incorporates value-reasoning and extends means-end reasoning. Surprisingly, with the notable exception of Clarke (1985), the above authors only mention that there are forms of practical reasoning different from means-end reasoning but do not specify which these are. Moreover, Searle (2001) argues that practical reason is more than just (short-term) means-end reasoning and criticizes the idea that rational agents are essentially goal-driven, reducing any rationality in action to means-end reasoning. See also Dancy (2018) for a critical discussion of this focus on means-end reasoning.

6 Others, who argue for intentional states as conclusions of practical reasoning, consider the necessitating conclusion ‘I should do X’ as a possible instance of such an intentional state, see for instance Lewiński (2021). We make a distinction between the two types of conclusions since those arguing for a normative conclusion often consider this as the only kind of conclusion.

7 Some of these accounts take the conclusion of (s3) as invalid (e.g., Clarke 1985), whereas others claim that the ‘must’ in the conclusion is a logical or epistemic must, rather than a practical must (e.g., von Wright 1963).

8 Such hypothetical statements are known as *anankastic* conditionals. These conditionals have been considered as *enthymemes* of practical inferences, but contemporary linguists take them as conditionals in their own right, posing essential difficulties for the evaluation of the involved modals in the conditional. See the work by Condoravdi and Lauer (2016) for an overview.

9 See also Corredor (2023) who, in this special issue, discusses the nature of the conclusion of practical argument.

10 Lewiński (2021) adopts the distinction between internal versus external. We adopt the term ‘communication’ for PA instead of externalized PR to stress the interaction between arguer and addressee, which we believe is more than just externalized reasoning on the part of the arguer. We come back to this in Sect. 5.

11 Clarke’s discussion of second- and third-person perspective practical inferences has some similarities with Gauthier’s (1963) account of taking a second-person normative statement to convey advice, as well as with argumentation in general, taking the aim of second- and third-person practical inferences to be to “persuade the hearer to perform the action or inducing a belief in the hearer in the truth of the proposition expressed’’ (Clarke 1985, p. 64).

12 We remark in passing that, when the arguer purports a speech act ‘You must fill in this form!’, this may require what is called ‘audience uptake’ in speech act theory (Green 2010). Supposing that the arguer does not have any commanding authority over the addressee, the remark is only normatively binding if the addressee endorses the reasoning themselves. Without uptake, there is merely an act of speech and we speak of a misfire instead (Austin 1962).

13 Of course, throughout a discussion other problems may be introduced that bear the potential of generating practical commitments. For instance, an argument ‘Precarious groups in society are in trouble, hence the government should do something about it’ put forward by the arguer may receive a reply from the addressee ‘Well, then you should start a petition’, thus introducing a sub-problem with the arguer as problem solver.
